# Stricture of the Male Urethra and Some Forms of Neuroses

**Published:** 1890-06

**Authors:** R. O. Engram

**Affiliations:** Member of the Academy of National Sciences; Member American Medical Association; Vice-President of the Medical Association of Georgia; Formerly Resident Physician of Philadelphia Hospital, etc.


### STRICTURE OF THE MALE URETHRA AND SOME FORMS OF NEUROSES.

BY R. O. ENGRAM, A. M., M. D.,

Member of the Academy of National Sciences; Member American Medical Association; Vice-President of the Medical Association of Georgia; Formerly Resident Physician of Philadelphia Hospital, etc.

It is not my purpose to enter into an extended notation of the various forms of nervous phenomena that are developed by the stricture of the male urethra, for to do so would require much time.

I will briefly note two or three, which, to a greater or less extent, are quite frequently present, but no doubt are attributed most frequently to other than the right cause.

Blisters, hypophosphites, strichnia, belladonna, morphia, digitallis, cactus grandiflora, pepsine, electricity, porous plasters and counter-irritants, and all like applications and therapeutics, no doubt, have been woefully misapplied by being administered to suffering men, where a meatotome, urethratome or steel sound would have avoided months, perhaps years, of miserable mental and physical suffering.

I will first bring to your attention

### VARICOSE VEINS.

That stricture produces varicose veins of the lower extremities, I am now satisfied, after extended observation. I at one time labored under the impression that the varicose veins that were occasionally brought to my attention by my patients were the consequence of urethrotomy or the after dilation I practiced. That this may occasionally supervene upon the use of the sound, is probable; yet I now believe it to be more from resultant changes already produced by a diseased urethra, and only precipitated by dilation.

Varix following stricture, especially complicated with enlarged prostate, has been so frequently noted by me, that I make it a rule to inquire, before exploration, if there be varicose veins, and if consulted for varix, I inquire at once into the condition of the urethra; frequently I find the two associated together in the same person. Operation for the coarctation always facilitates the cure of the varix.

I now never operate for varix in the male without consulting the urethra; and never in females without consulting the bladder and organs of generation.

All surgeons who have much experience in the treatment of the male sexual organs are aware how frequently they encounter hemorrhoids in these troubles, and gynecologists appreciate the association of hemorrhoids with diseases of the uterus and urinary organs of women.

As to the causes of these varicose conditions, we may be warranted in assuming that the vaso-motor centers are very violently disturbed by the reflex vibration set up from coarctations occurring in the urethra, or other forms of urethral irritation. Such irritation through the pudic nerve causes spasms and dilatation of the veins, increased blood-pressure and impeded return blood-flow, if not stasis, temporarily complete, permitting a loss of elasticity of the muscular coats of the blood-vessels; dilatation, impaired vascular action, loss of proper nutrition of the parities of the vessels, and consequent varix.

ATAXIA.

Among other neuroses is a peculiar condition approaching ataxia. I formerly attributed the symptoms to a depressed form of spinal irritation, or neurosthenia, there being present a "nervous weakness," with pain in the lumbar and dorsal regions, with renewed tenderness over the spinous processes, accompanied with a burning tension, dyspnœa, oppression, palpitation and numbness of the scalp. On exercise, a weary, heavy sensation, with muscular pains arise, also excitable nervous system, with loss of virile powers, more or less complete, premature ejaculation, loss of will power, with deficient judgment and memory; at times excruciating pains in the back of the legs, and other neuroses that might lead us to believe that neurasthenia, more or less complete, might exist.

Ataxic symptoms are sometimes very prominent. I give a brief history of a case.

Mr. ——, æt. 49, had gonorrhœa some twelve years previous; drank freely. About a year previous to my connection with his case he had some failure of appetite, loss of flesh and irritability and loss of judgment and impairment of memory, which previously had been extraordinary; intelligence but little affected; became extremely restless; great pains in the calves of the legs, with marked sciatic pains; considerable hyperæsthia of the cutaneous surface over parts of the body and lower limbs; spinous process of lumbar regions very sensitive; excessive tension of limbs; ascent and descent of stairs could not be made without assistance; locomotion at night impossible without guidance of a servant, unless the moon shone brightly, then only indifferent locomotion; various dyspeptic symptoms arose and resisted treatment.

The attending physician attributed the condition to some form of paresis arising from excessive use of alcoholic stimulants.

After an extended treatment with strychnia faradisation and tonics, with no relief, the patient fell into my hands. He had all the enumerated symptoms, with *cincture feeling*, or constriction about the girdle. He says he feels as if he had an "anvil" in his stomach. The hypeaæsthesia of the surface of his body and lower limbs was so great that he would scarcely admit the pressure of examination. Deep pressure in either hypogastric region along the course of the lumbo-sacral cord and great sciatic caused excruciating pain. The stomach felt as hard to the touch as a cast-iron ball. The eyes had a bright watery stare—I might say brilliant stare—with dilated pupils contracting only hesitatingly to light.

When I inquired into his power of locomotion, he expressed his confidence freely in his ability to walk, nevertheless his gait was uncertain. On being blind-folded, locomotion was entirely prevented. I drew a ring three feet in diameter upon the carpet and requested him to walk out, or run out, if he could. He affirmed most confidently he could, if I would prevent his falling into the grate. On attempting to walk out, he made no progress, but alternately raised his feet up and down in a fair imitation of a raw militiaman's attempt at marking time. Imagine his great surprise on being unblind-folded at finding himself still in the ring.

Deep pressure in the perinæum elicited great pain, so much that I feared deep-seated abscess. On the introduction of No. 8 exploratory bougie which was with difficulty accomplished, convulsions seemed imminent in spite of cocaine, previously used, spasmodic contractions of the fingers and toes were violent and persisted for an hour, so great was the hyperæsthesia of the urethra. A brownish discharge was brought along with the withdrawn sound. Exploration revealed coractation No. 8, at two and one-half inches from meatus, which patient had never suspected, nor which had given no inconvenience except an occasional irritable condition of kidneys and bladder, so he said. He had not slept for weeks, except under anodynes.

Dilating urethrotomy, in twenty-four hours, greatly removed the locomotor ataxic symptoms, much of the hyperæsthesia had passed away, the *cincture* had grown less distressing, the "anvil" in the stomach had grown smaller ; and prolonged and refreshing sleep supervened; all without a single dose of medicine.

This operation was performed three months ago (January, 1890.) The patient formerly weighed 240 pounds, had reduced to 169, now weighs 205 pounds with all of his troubles dissisipated.

This is, of course, an aggravated case. I have observed varying degrees of these ataxic symptoms, from slightly unsteady gait through varying stages, including syncope, intractacable reflex dyspepsia, palpitation, basilar, headache and a long train of reflex irritations.

I now call to mind two patients, one of whom, upon the least excitement, would fall into a most alarming case of vertigo. The other, of very high-strung irritable temper, nervous and irascible to a very distressing degree, would go into violent tetanic spasms of the muscles of the hands, as is sometimes observed in hystei o-epilepsy, upon having his feelings wounded, which could be easily done upon the least provocation.

Both of these patients suffered intense pain along the sciatic nerve, urethrotomy and galvanism gave them prompt relief, with no return of their troubles, now two and five years past.

TROPHIC AFFECTION OF THE SKIN.

Neuro-dermatitis, I am convinced, is a frequent result of urethral irritation, and especially herpetic eruptions of the penis, and more constantly of the prepuce on the inner surface or at the junction of the skin and mucous membrane. Why not? Herpes of other parts of the body have long been looked upon as of neurotic origin.

Few writers now deny that zona and zosta are from some unhealthy excitation of the nervous system, still none has inquired into the fact that urethral irritation might set up these trophic disturbances of the skin in widely divergent parts of the body.

"Herpes præputiallis," says Pepper, "is met with in persons who have been affected with gonorrhœa, chancroids, and chancres, and a long prepuce is a predisposing factor."

He lays stress upon those who have been affected with gonorrhoea. Truly they are more subject to urethral irritation, and stricture; hence, to Herpes praeputiallis. Professor Felix Von Neimyer passes over the subject of stricture with a bare notice, not mentioning such a thing as reflex. Abner Post cleaves to the idea of nerve irritation. Muriac looks upon it as neuralgic. Unna attributed it to excessive congestion of the genitals. Tilbury Fox says, "Herpes' praeputiallis has come on after emission, after connection regularly, so as to leave no doubt as to the co-relation; and after other irritations of the urethral mucous membrane.

Prof. T. Holmes, surgeon to St. George Hospital, dismisses the subject of Herpes' praeputiallis, with scarcely a notice, except to speak of it as being easily cured, and to draw its diagnostic difference from incipient chancre. Prof. S. D. Gross attributes the disease to friction, want of cleanliness and disorders of the digestive organs.

So none have attributed to Herpes, an origin from stricture. It is true that Tilbury Fox borders upon the subject, when he says, "After other irritation of the urethral mucous membrane." Abner Post says nerve irritation; but is not specific; does not name the source of irritation. Muriac says neuralgia. He fails to say whether neuralgia is set up by miasm, cold, indigestion, or what form of nerve irritant.

To illlustrate, I cite a case:

G., age 43, white, married, no history of syphilis, digestion fair. Had gonorrhœa fourteen years ago. Herpes upon the glans. Prepuce thickened and sensitive. Pain from the eruption sometimes very severe. Marked sciatic pains with weary, distressed feelings in the back of the legs, Herpes upon the prepuce and glans penis of twelve years' duration. Had exhausted the materia medica professional and lay; taken anti-syphilitics, anti-neuralgics, hypophosphites, strychnine, arsenic ointments and plasters and every conceivable officinal and patent blood purifier known and obtainable, without the least impression upon the disease.

I saw this patient six years ago in consultation and advised the importance of urethral exploration, but it was not encouraged by the attendant nor patient. I lost sight of the man until last September, when he consulted me in my office. I reiterated my former opinion, and upon exploration verified it by discovering a structure No. 17 at one inch from the meatus, and another at five inches, No. 20. I performed dilating urethrotomy up to No. 31; and dilated with conical steel bougies for one month, used no medication external or internal, and in twenty days, for the first time an eruption of twelve years standing had vanished. In two months all inflammatory deposit was gone, and parts looked normal and healthy. I had a letter from him on 9th of April this year, (1890) stating that he remains well.

Another very interesting case I had in 1875, in a young gentleman who had an herpatic eruption upon the glans, and upon the prepuce; externally all over the penis and left scrotum, around the navel, on the throat just covering the pomum adamie. The internal throat, and especially the tonsils were herpatic, an eruption extended along the course of the popliteal nerve for about three inches. Occasionally the eruption would diminish, but not disappear; but on excessive venereal indulgence, the eruption would be aggravated.

This patient was a midshipman in the Navy, and on an extended cruise the eruption very perceptibly faded, and he hoped ultimate relief was close at hand; but on his return, while stationed at Annapolis, Md., he indulged in an excessive venereal escapade, which brought back his old trouble so severely that he applied for and obtained a furlough, when he fell into my hands, and upon exploration I found a coarctation No. 22, three inches from the meatus. Dilating urethrotomy up to 32 entirely relieved him in a few weeks. The last I heard of him, two years ago, he had suffered no return of his trouble.

Recurring to the involvement of the tonsils in this case, I will say in conclusion, that I have frequently noted throat complications of various kinds in urethral irritation, especially in young subjects. The frequency of Hypertrophy of the tonsils and elongated prepuce coincidently, in little boys, has led me to question if they are not inter-dependent.

Hypertrophy of the tonsils in females where the uterus is involved has frequently attracted my attention. I do not now call to mind a single incidence where I have been consulted for hypertrophic tonsilitis in young girls who did not develop some uterine complication when womanhood was reached, or some intractable menstrual disorder at puberty, or following soon thereon. I cannot attribute this to struma, except the seeds of struma were sown primarily in the undeveloped, immature uterus; for I have yet to find a single case of enlarged tonsils in young girls that did not improve when menstruation was normally established, without regard to therapeutic applications, whether directed to the tonsils or not. Relief of menstrual and uterine troubles notes a period of improvement in hypertrophy of the tonsils of the female, so relief of urethral and præputial irritations mark a period of improvement in hypertrophy of the tonsils in the male.

---

The February issue of *Babyhood* states that in an Edinburgh professional journal a simple and ingenious contrivance is mentioned, to admit of the continuous inhalation of steam fumes by patients suffering from diphtheria. This is nothing more than the fixing of an open umbrella to the bed, or suspending it from the ceiling, and throwing over this a large sheet, which, falling in a tent above the patient, will surround him with the atmosphere of steam. The steam is supplied by a pipe connection with a kettle or other boiling contrivance that passes beneath the tent. The suggestion is so admirable and feasible that we are sure it will be welcomed by many physicians, who are sometimes at a loss, in the absence of especially devised contrivances, to know how to effect with simple means the end desired in such cases.—*College and Climical Record.*

